# Supplementary material for: WordCommentsAnalyzer: A windows software tool for qualitative research
Source: F1000Res. 2018 Sep 4;7:536. Originally published 2018 May 3. [Version 2] doi: 10.12688/f1000research.14819.2 (PMC6137411; doi:10.12688/f1000research.14819.2)
Supplement: Supplementary file 3 [file f1000research-7-17614-s0002.tgz › b2a79496-4ada-47c4-9682-a99ce867836d.zip!/Eight Iranian Health Journals/Yafteh _56.docx]

© Yafteh  . This work is licensed under CC BY 4.0 (https://creativecommons.org/licenses/by/4.0/).

Volume & Issue: دوره 7 - شماره 1

Title: آلکالوئید استروئید های مجزا شده از برگ زیتون آسیب ناشی از ایسکمی مغزی در رت را کاهش می دهند

http://yafte.lums.ac.ir/article-1-1062-fa.html

مقدمه: آلکالوئید استروئید های مجزا شده از گونه های گیاهی متعدد به علت خواص فارماکولوژیک متنوع توجه زیادی را به خود جلب کرده اند . در این تحقیق هدف بر این بوده است تا اثرات آلکالوئید استروئید های مجزا شده از برگ زیتون بر ایسکمی مغزی در رت بررسی شود نتیجه گیری: این نتایج نشان می دهند که آلکالوئید استروئیدهای مجزا شده از برگ زیتون اثرات محافظتی روی آسیب ایسکمی مغزی –خونرسانی مجدد در رت دارند و مکانیسم آن ممکن است با کاهش دادن تجمع کلسیم و پراکسیداسیون لیپیدها ارتباط داشته باشد. یافته ها: تزریق داخل وریدی آلکالوئید استروئید ها (mg/kg 5/0، 1، 5/1) اندکس سکته را کاهش ، دامنه الکتروانسفالوگرام (EEG) راافزایش و غلظت لیپید پراکسیداز (LPO) و محتوی کلسیم قشر مغز را به روش وابسته به دوز کاهش دادند؛ اما هیچ تفاوت معنی داری در غلظت سدیم و محتوی آب قشر مغز مشاهده نشد. مواد و روش ها: در این مطالعه آزمایشی 48 رت نژاد ویستار به شش گروه 8 تایی تقسیم شدند و ایسکمی مغزی بوسیله مسدود کردن شریانهای کاروتید و مهره ای دو طرف به مدت 10 دقیقه ایجاد شد و سپس به مدت 5 روز بعد از باز شدن شریانها مورد بررسی قرار گرفتند .

Title: سندروم های حاد کرونر و فاکتورهای کمپلمان

http://yafte.lums.ac.ir/article-1-1065-fa.html

مقدمه: بیماری عروق کرونر از شایع ترین و مهم ترین علل مرگ در جهان است. زمینه ساز اصلی بیماری، آترواسکلروز عروق کرونراست. این بیماریهای عروق کرونردارای طیف وسیعی است که در یک سر طیف ایسکمی بدون علامت و در سر دیگر طیف، مرگ ناگهانی است. هدف این مطالعه بررسی تفاوت ها و مقایسه تغییرات اجزای c3 و c4 کمپلمان در این دو بیماری است. تا شاید بتوان از این اجزا به عنوان شاخص هایی برای تعیین پیش آگهی و عوارض حاد آنها استفاده نمود. مواد و روش ها: در این مطالعه مقطعی 30 بیمار از هر گروه بیماران دچار انفارکتوس حاد قلبی و آنژین صدری ناپایدار که در بخش مراقبت های ویژه قلبی بستری شده بودند، با 30 فرد سالم داوطلب به عنوان گروه کنترل مقایسه شدند. یافته های بالینی و آزمایشگاهی در زمان بستری، 12 ساعت، 24 ساعت و 48 ساعت بعد جمع آوری شد. از همه بیماران نوار قلب در بدو ورود و روزانه گرفته شد، اکوکاردیوگرافی به عمل آمد و از همه بیماران نمونه خون برای تعیین سطح سرمی کمپلمان گرفته شد. بیماران تا 14 روز پس از ترخیص پیگیری شدند.نتیجه گیری: در گروه بیماران مبتلا به انفارکتوس حاد قلبی سطح سرمی اجزای کمپمان با بروز نارسایی قلب و انفارکتوس، موج Q و مرگ و میر ارتباط داشت؛ ولی در بیماران مبتلا به آنژین صدری ناپایدار تنها بیانگر فعال شدن این سیتم است و فقط سطح سرمی C3 با میزان قدرت انقباضی قلب ارتباط داشت. یافته ها: در بررسی انجام شده سیستم کمپلمان در هر دو گروه بیماران مبتلا به انفارکتوس حاد و آنژین صدری ناپایدار فعال شده بود. در گروه بیماران مبتلا به انفارکتوس حاد میزان فعالیت شدیدتر از گروه بیماران مبتلا به آنژین صدری ناپایدار بود (01/0p<). در بیماران مبتلا به انفارکتوس حاد و نارسایی قلبی متعاقب آن و در بیماران با انفارکتوس حاد با موج Q بالاتر از بقیه موارد بود (01/0p<). در بیماران با انفارکتوس حاد سطح سرمی اجزا ی کمپلمان به طور مستقیم با بالاترین سطح Cratin phospho kinas (CPK) و به طور معکوس با کسر تخلیه بطنی مرتبط بود (71/0r=) و از طرفی افزایش سطح سرمی کمپلمان به طور مستقیم با افزایش مرگ و میر بیماران ارتباط داشت. در بیماران مبتلا به آنژین صدری ناپایدار بین سطح سرمی C3 با کسر تخلیه بطنی ارتباط معنی دار وجود داشت (71/0r=). مقایسه سطح سرمی کمپلمان دو گروه انفارکتوس حاد و آنژین صدری ناپایدار با گروه کنترل اختلاف معنی داری را نشان داد (01/0p<).

Title: بررسی میزان شیوع رتینوپاتی وعوامل مؤثر بر آن در بیماران دیابتی بستری شده در بیمارستان بقیه‌الله الاعظم «عج» تهران طی سال های 1377 تا 1381

http://yafte.lums.ac.ir/article-1-1066-fa.html

مقدمه: رتینوپاتی یکی از عوارض مزمن دیابت است که شایع‌ترین علت نابینایی درسنین 74ـ20 سالگی در کشورهای پیشرفته است.گرچه عامل اصلی ایجاد رتینوپاتی، هیپرگلیسمی است؛ اما عوامل دیگری نیز در بروز آن نقش دارد. هدف از انجام این مطالعه تعیین میزان شیوع رتینوپاتی در بیماران دیابتی وشناسایی عوامل مؤثر بر آن در یک جمعیت ایرانی بود. مواد و روش ها: این مطالعه از نوع توصیفی به روش مقطعی (Cross-sectional) بود. جامعه مطالعه شامل بیماران دیابتی مراجعه کننده به بیمارستان بقیه ا...(عج) بود که به دلایل مختلف در بخش داخلی بستری شده بودند. 300 بیمار با استفاده از فرمول برآورد اندازه نمونه برای نسبتها به شیوه نمونه گیری تصادفی انتخاب شدند ونتایج با آزمون‌های و مجذور کای و آزمون دقیق فیشر تحلیل گردید.نتیجه گیری: با توجه به نتایج این مطالعه توصیه می گردد بررسی شبکیه چشم در ارزیابی بیماران دیابتی همیشه مدنظر باشد و همچنین سطح سرمی قند در حد طبیعی نگهداری شود. به دلیل وجود ارتباط بین هایپر لیپیدمی ، استعمال سیگار و فشار خون با رتینوپاتی پیشنهاد می گردد که این سه عامل نیز در بیماران دیابتی مورد توجه جدی قرار گیرند. یافته ها: میزان شیوع رتینوپاتی 6/56 درصد تعیین شد. میانگین سن افراد مبتلا به رتینوپاتی 3/60 سال(انحراف معیار=10) و میانگین سابقه ابتلا به دیابت 15 سال(انحراف معیار=1/7) بود. فراوانی نسبی رتینوپاتی غیرتزایدی Non prolifrative diabetic retinopathy (NPDR) و تزایدی (PDR) Prolifrative diabetic retiropathy به ترتیب 8/52 درصد و 2/47 درصد بود. این مطالعه نشان داد که ارتباط بین رتینوپاتی و طول مدت ابتلا به دیابت ، سن بیماران، سطح بالای قند خون ناشتا، سوابق هایپرلیپدمی، سیگار کشیدن و بیماری ایسکمیک قلبی از نظر آماری معنی دار است. (001/0P<). ارتباط بین رتینوپاتی وسابقه پرفشاری خون نیز از نظر آماری معنی داربود (05/0P<) . دراین مطالعه ارتباط بین رتینوپاتی و جنس،نوع داروی مصرفی و نیزسابقه سکته مغزی از نظرآماری معنی دارنبود.

Title: تأثیر سدیم والپروات در کنترل علایم ترک در بیماران وابسته به تریاک

http://yafte.lums.ac.ir/article-1-1067-fa.html

مقدمه: تحریک کننده های گاماآمینوبوتیریک اسید (GABA) در کاهش علائم ترک بنزودیازپین ها، خواب آورها و الکل و کاهش مصرف اختیاری کوکائین مؤثر بوده اند. والپروات سدیم دارویی GABAergic است که در کنترل دردهای نوروپاتیک و همچنین در کنترل علائم ترک بنزودیازپین ها و الکل مؤثر بوده است. مطالعه حاضر به منظور بررسی اثر این دارو در کنترل علائم ترک وابستگی به تریاک و تأثیر آن در کاهش مدت زمان بازگیری از متادون انجام شد. مواد و روش ها: تعداد 60 بیمار مراجعه کننده که مصرف کننده تریاک و دارای معیارهای راهنمای تشخیصی و آمار اختلالات روانی، بازنگری چهارم DSMI-V از نظر وابستگی به تریاک بودند با رعایت معیارهای شمول و حذف به طور تصادفی به دو گروه تقسیم شده و بصورت دوسوکور تحت درمان با سدیم والپروات یا پلاسبو علاوه بر داروهای مشترک (‌متادون وکلونیدین) قرار گرفتند. شدت علائم ترک در روزهای 2،5،9،12،19،26 با مقیاس Modified Short Opioid Withdrawal Scale مورد ارزیابی و نتایج دو گروه از طریق T-Test با هم مقایسه گردید.یافته ها: میانگین نمره علائم فیزیکی سندرم ترک در دو گروه در روزهای مختلف تفاوت معنی داری نداشت؛ اما در کنترل علائم روانی، سدیم والپروات نسبت به پلاسبو تأثیر قابل ملاحظه ای داشت. نتیجه گیری: سدیم والپروات ممکن است به عنوان داروی کمکی در کنترل علائم روانی سندرم ترک اعتیاد تأثیر مثبتی داشته باشد. مطالعات بزرگ تر و طولانی تربرای تأیید یافته های فوق و بررسی میزان تأثیر داروی سدیم والپروات در جلوگیری از عود وابستگی به تریاک پیشنهاد می شود.

Title: تعیین سطح سرمی دیگوکسین در بیماران با نارسایی قلبی

http://yafte.lums.ac.ir/article-1-1068-fa.html

مقدمه: نارسایی قلبی یکی از بیماری های شایع قلبی و عروقی است و داروی دیگوکسین در فهرست درمان های دارویی لازم است. این دارو با توجه به تأثیر مثبت آن بر نارسایی قلب متاسفانه سطح سرمی درمانی و توکسیک آن در افراد مختلف، متفاوت و بسیار به هم نزدیک می باشد. هدف از انجام این مطالعه بررسی سطح سرمی دیگوکسین در بیماران دچار نارسایی قلبی بود. مواد و روش ها: در این مطالعه توصیفی - مقطعی 31 بیمار با نارسایی قلبی و مصرف کننده دیگوکسین، مراجعه کننده به درمانگاه بیمارستان تأمین اجتماعی و شهدای عشایر خرم آباد، مورد بررسی قرار گرفتند. برخی عوامل مهم و دخیل در تعیین سمیت دیگوکسین از قبیل سن، جنس، کراتینین، کلیرنس کراتینین، تخلیه قلبی، سطح اوره، پتاسیم، کلسیم و دیگوکسین، اندازه گیری شد. نتیجه گیری: به نظر می رسد جهت پیشگیری ازافزایش سطح دیگوکسین سرم و مسمومیت باآن کنترل مداوم سطح سرمی این دارو به صورت اندازه گیری سرمی و یا با توجه به آن کلیرنس امری ضروری است. یافته ها: تحلیل آماری نشان دادکه با افزایش سن سطح سرمی، دیگوکسین افزایش می یابد (01/0p<) . افزایش سطح سرمی دیگوکسین با سطح سرمی اوره، کراتینین و پتاسیم ارتباط داشت (01/0p<).

Title: بررسی آنفارکت هموراژیک در بیماران سکته مغزی

http://yafte.lums.ac.ir/article-1-1069-fa.html

مقدمه: ترانسفورماسیون هموراژیک از یافته های مهم سکته (انفارکتوس) مغزی است که در سکته های ناشی از آمبولی قلبی بیشتر گزارش شده است . گرچه منجر به بدتر شدن وضعیت بالینی بیمار نمی شود؛ ولی تصمیم گیری در مورد تجویز داروهای ضد انعقادی را در این بیماران دشوار می سازد . هدف از انجام این مطالعه، بررسی فراوانی نسبی و علت سکته هموراژیک مغز و تأثیر آن بر روند بهبودی در بیماران بستری شده در بیمارستان ولیعصر (عج) خراسان بود. مواد و روش ها: این مطالعه توصیفی ـ مقطعی از 1379 تا 1382 بر روی تمامی بیماران بستری شده با تشخیص سکته مغزی در بیمارستان دانشگاهی ولی عصر (عج) خراسان انجام شد. تشخیص آنفارکت هموراژیک با سی تی اسکن مغز 24 تا 48 ساعت بعد از شروع علائم سکته و تعیین قلمرو عروقی مبتلا نیز با استفاده از نقشه های توپوگرافیک مغز در سی تی اسکن صورت گرفت . وضعیت بالینی بیماران دچار آنفارکت هموراژیک هر 12 ساعت در مدت بستری ارزیابی شد. علت سکته مغزی در بیماران با یافته های بالینی ، سی تی اسکن ، آزمایشات بیوشیمی خون ، نوار قلب ، اکوکاردیوگرافی ترانس توراسیک و سونوگرافی دوپلکس عروق کاروتید و داپلر ترانس کرانیال مشخص شد. نتیجه گیری: آمبولی با منشأ قلبی علت معمول آنفارکت هموراژیک مغز نیست بنابراین به نظر می رسد فرضیه آدامز و فیشر عمومیت ندارد. ترانسفورماسیون هموراژیک در موارد کمی منجر به بدتر شدن وضعیت بالینی بیماران می گردد. یافته ها: از 302 بیمار بررسی شده 26 نفر(6/8%) شامل 13 زن و 13 مرد ترانسفورماسیون هموراژیک داشتند . در 88% قلمرو عروقی شریان مغزی میانی آنفارکت هموراژیک مشاهده شد. به نحوی که قلمرو خون رسانی شریان لنتیکولواستریت در 46% بیماران به تنهایی و در23% موارد همراه با سایر شاخه های شریان مغزی میانی دچار سکته بود. وضعیت بالینی به دنبال ترانسفورماسیون هموراژیک در4 /15% بیماران رو به وخامت، در 5 /38% موارد رو به بهبود و در 46 % نیز بدون تغییر بود. آمبولی با منشأ قلبی 6/34 % علت های آنفارکت هموراژیک مغز در بیماران بود.

Title: شیوع میگرن در دانش آموزان دبیرستانی شهر خرم آباد

http://yafte.lums.ac.ir/article-1-1070-fa.html

مقدمه: میگرن شایع ترین نوع سردرد در سراسر دنیا است و یکی از مهم ترین علل مراجعه بیماران به مطب های تخصصی اعصاب را تشکیل می دهد. هدف از انجام این مطالعه بررسی شیوع میگرن در دانش آموزان دبیرستانی خرم آباد بود. مواد و روش ها: این پژوهش یک مطالعه توصیفی بود که تعداد 2213 نفر از دانش آموزان دبیرستانی که طی سال تحصیلی 81-80 مشغول به تحصیل بودند به صورت تصادفی چند مرحله ای به عنوان نمونه انتخاب شدند. ابزار گردآوری اطلاعات پرسشنامه ای مشتمل بر دو بخش بود. بخش اول شامل متغیرهای زمینه ای (سن، جنس و…) بود و بخش دوم شامل متغیرهای اصلی تحقیق از جمله سردرد، تهوع ، استفراغ، زمان شروع سردرد، وجود علائم بینایی و… بود. اطلاعات به دست آمده با استفاده از نرم افزار SPSS 9.0 تحلیل شدند. نتیجه گیری: با توجه به شیوع بالای سردردها بین تمونه مطالعه شده و مشکلات متعددی که سردرد به دنبال دارد لزوم توجه به درمان انواع سردرد و رفع آنها از اهمیت زیادی دارد . یافته ها: نتایج بدست آمده نشان داد که 46/44 درصد از افراد مورد مطالعه دارای سردرد بودند. دانش آموزان دختر (9/68%) بیشتر از دانش آموزان پسر (09/31%) مبتلا به سردرد بودند. شایع ترین نوع میگرن، میگرن شایع (44/65%) بود. 4/0% از سردردها از نوع میگرن افتالموپلژیک بودند. 53/32% از کل سردردها را سردرد تنشی تشکیل می داد.

Title: وضعیت ویتامین A سرم در بانوان مصرف کننده قرص های ضد بارداری خوراکی

http://yafte.lums.ac.ir/article-1-1071-fa.html

مقدمه: ویتامین A یکی از مهمترین سیستم های دفاعی آنتی اکسیدان غیر آنزیمی به شمار می رود و یک عامل رژیمی مهم برای کاهش وقوع بیماری های قلبی- عروقی است. مطالعه حاضر با هدف بررسی وضعیت سرمی ویتامین A در بانوان مصرف کننده قرص های ضد بارداری خوراکی (OCs) با دوز پائین محتوی mg15/0 لونورژسترول و mg03/0 اتینیل استرادیول و نیز در بانوان یائسه تحت درمان جایگزینی هورمون (HRT) و مقایسه آنها با گروه های شاهد انجام گرفت. مواد و روش ها: در این مطالعه مورد ـ شاهدی، 139 نفر از زنان سالم غیر باردار و غیر شیرده با میانگین سنی 32 سال (70 نفر استفاده کننده از OCs و 69 نفر از غیر کاربران OCs) و 60 نفر زنان یائسه سالم با میانگین سنی 51 سال (30 نفر تحت درمان HRT و 30 نفر بدون درمان HRT) با استفاده از روش های بیوشیمیایی، تن سنجی و مصاحبه مورد مطالعه قرار شدند. داده ها با استفاده از آزمون t و x2 تجزیه و تحلیل شد. نتیجه گیری: استفاده ازOCs و تغییرات هورمونی ناشی از آن عاملی مؤثر بر افزایش سطح ویتامین A سرم در بانوان سنین باروری است. درمان با HRT با ترکیب به کار رفته تغییری بر وضع این ویتامین در گروه یائسه ایجاد نکرده است. یافته ها: بر اساس یافته های بدست آمده، اختلاف آماری معنی داری از نظر میانگین BMI، تعداد بارداری، دریافت ویتامین A و فیبر بین دو گروه OCs و غیر OCs و نیز بین دو گروه HRT و غیر HRT وجود نداشت. در گروه مصرف کننده OCs میانگین سطح ویتامین A سرم به طور معنی داری (001/0p<) بیش از گروه غیر OCs (µg/dl56/23±97/79 در مقابل µg/dl 15/22±56/64) بود. بین میانگین سطح سرمی ویتامین A سرم در دو گروه HRT و غیر HRT اختلاف آماری معنی داری مشاهده نشد(به ترتیب µg/dl 91/15±76/71 و µg/dl 39/22±65/67). با استفاده از آزمون x2 رابطه آماری معنی داری بین وضعیت ویتامین A سرم و استفاده از OCs به دست آمد (004/0p<). این آزمون در گروه های یائسه رابطه معنی داری نشان نداد.
